# Supplementary material for: APOE-ε4 and BIN1 increase risk of Alzheimer’s disease pathology but not specifically of Lewy body pathology
Source: Acta Neuropathol Commun. 2023 Sep 12;11:149. doi: 10.1186/s40478-023-01626-6 (PMC10496176; doi:10.1186/s40478-023-01626-6)
Supplement: Supplementary file 1 — Additional file 1. Supplementary Materials [file 40478_2023_1626_MOESM1_ESM.pdf]

Supplementary Materials

APOE-ε4 and *BIN1* increase risk of Alzheimer's disease pathology but not specifically of Lewy body pathology

Seth Talyansky[1,†], Yann Le Guen[1,2,†,*], Nandita Kasireddy[1], Michael E. Belloy[1], and Michael D. Greicius[1]

[1]Department of Neurology and Neurological Sciences, Stanford University, Stanford, CA, USA

[2]Institut du Cerveau – Paris Brain Institute – ICM, Paris, France

[†]Equal contributor

[*]Correspondence:

Department of Neurology and Neurological Sciences

Stanford University

290 Jane Stanford Way, E265, CA 94305-5090

Email: yleguen@stanford.edu

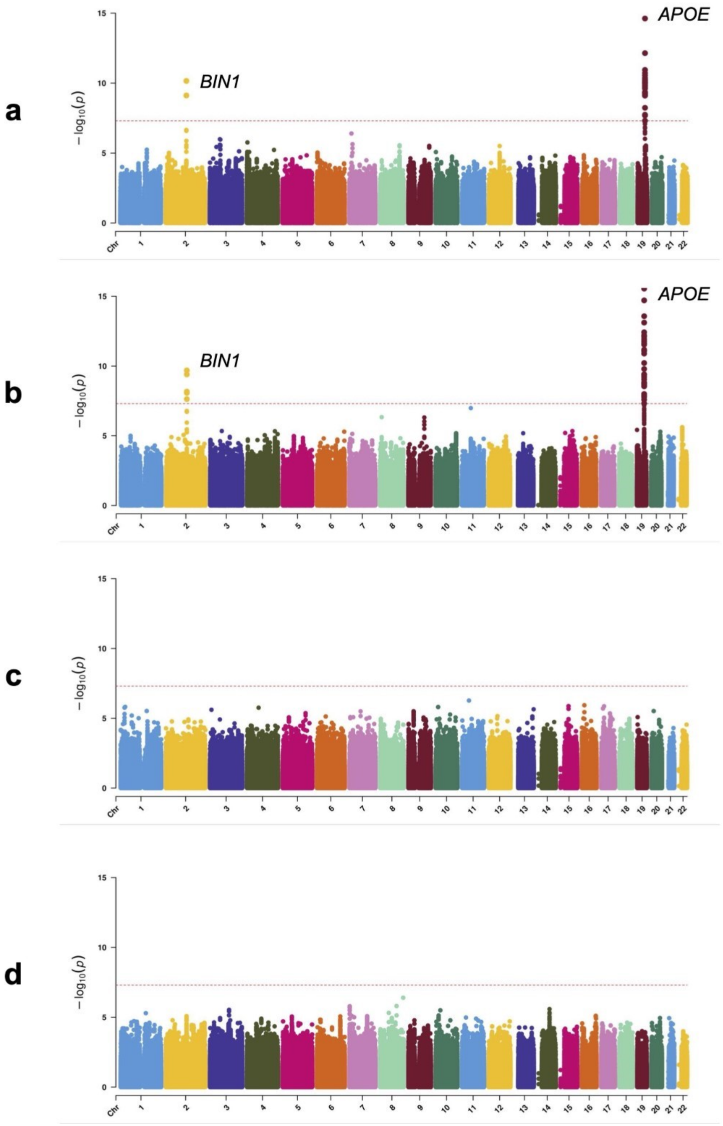

Supplementary Figure 1. Manhattan plots of genetic association with pathology classes in the subset of NACC individuals. A. Association with $AD^{+}LB^{+}$ pathology versus $AD^{-}LB^{-}$ pathology. **B.** Association with $AD^{+}LB^{-}$ pathology versus $AD^{-}LB^{-}$ pathology. **C.** Association with $AD^{-}LB^{+}$ pathology versus $AD^{-}LB^{-}$ pathology. **D.** Association with $AD^{+}LB^{+}$ pathology versus $AD^{+}LB^{-}$ pathology.

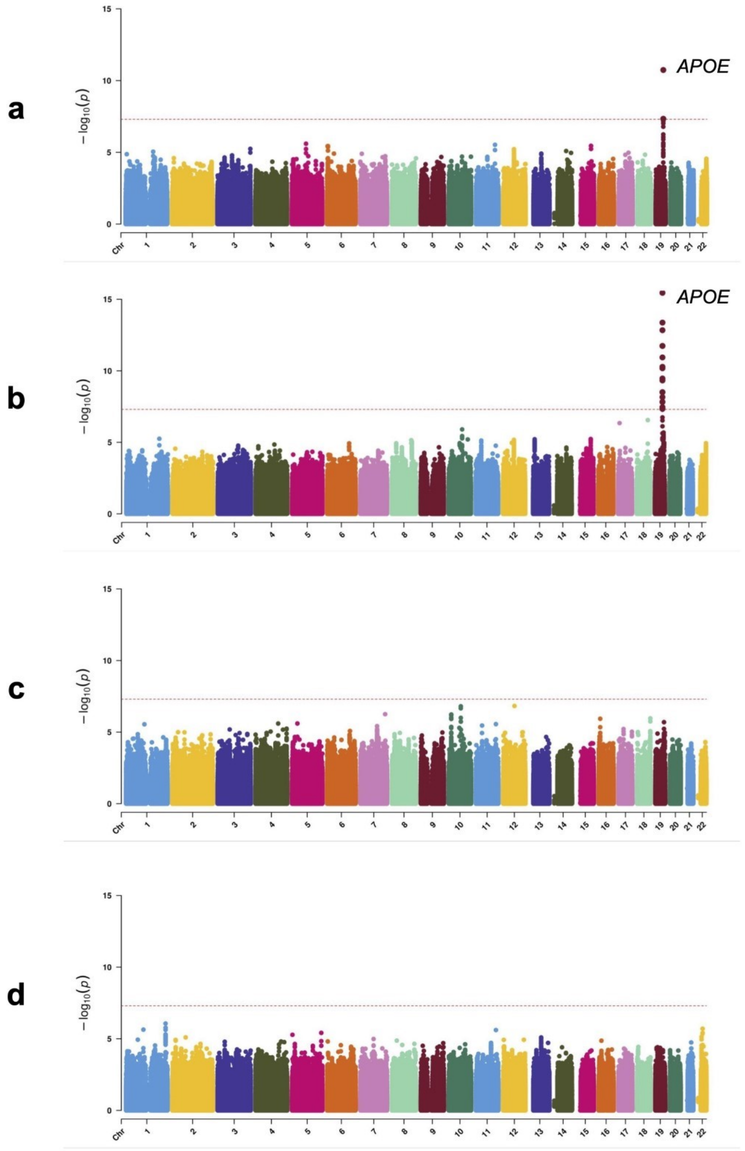

Supplementary Figure 2. Manhattan plots of genetic association with pathology classes in the subset of Rush individuals. A. Association with $AD^{+}LB^{+}$ pathology versus $AD^{-}LB^{-}$ pathology. **B.** Association with $AD^{+}LB^{-}$ pathology versus $AD^{-}LB^{-}$ pathology. **C.** Association with $AD^{-}LB^{+}$ pathology versus $AD^{-}LB^{-}$ pathology. **D.** Association with $AD^{+}LB^{+}$ pathology versus $AD^{+}LB^{-}$ pathology.

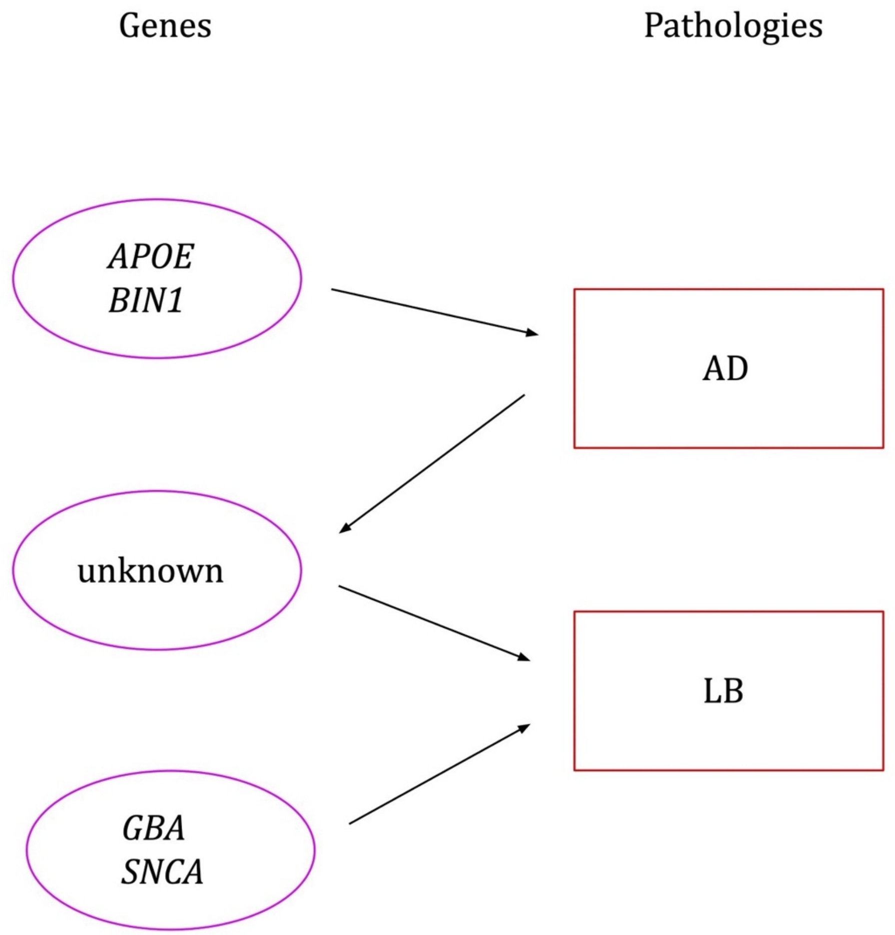

Supplementary Figure 3. A candidate genetic model of AD and LB pathology. *APOE* and *BIN1,* among other genes (see *e.g.,* Bellenguez *et al.*), drive AD pathology. AD pathology primes other, unknown genes to drive LB pathology. In a separate mechanism not involving AD pathology, *GBA* and *SCNA,* among other genes (see *e.g.,* Chang *et al.* (2017) and Nalls *et al.*), drive LB pathology. Notably, the set of genes including *APOE* and *BIN1* drives LB pathology only by way of AD pathology and does not affect the likelihood of LB pathology after the step of AD pathogenesis; this risk is determined by as yet unidentified genes.

Supplementary Table 1. Comparison of pathologically assessed proportion of cohort and sample size per pathology group between reference studies [52, 2, 10, 14, 24, 18, 46, 48, 13, 28] and ours. For Beecham *et al.,* Robinson *et al.,* and Sabir *et al.,* we consider only analyses of *APOE*-*ε*4-associated risk for LB pathology or dementia [2, 46, 48]. For Guerreiro *et al.,* we describe the larger discovery cohort [24]. If a study analyzed a cohort both with and without including certain clinically assessed subjects, we consider the analysis with the fewest clinically assessed individuals for which the effect size of *APOE*-*ε*4 and/or the top variant on *BIN1* is reported.

Study	Total size	# subjects clinically evaluated only	# pathologically confirmed AD$^-$LB$^-$	# pathologically confirmed AD$^+$LB$^+$	# pathologically confirmed AD$^+$LB$^-$	# pathologically confirmed AD$^-$LB$^+$	# pathologically confirmed LB$^{-\,a}$	# pathologically confirmed LB$^{+\,a}$
**Tsuang *et al.* (2013)**	640	0 (0%)	269	224	244	91	N/A	N/A
**Beecham *et al.* (2014)**	3,526	0 (0%)	?	?	?	?	2,391	1,135
**Bras *et al.* (2014)**	3,291	2,624 (80%)	0	?	0	?	0	667
**Chung *et al.* (2015)**	531	0 (0%)	0	215	316	0	N/A	N/A
**Dickson *et al.* (2018)**	1,310	660 (50%)	0	491^b (10+27+115+19 +111+209)	0	159^b (46+80+33)	N/A	N/A
**Guerreiro *et al.* (2018)**	5,007	4,033 (81%)	0	?	0	?	0	974
**Robinson *et al.* (2018)**	346	0 (0%)	0	226^b (19+111+10+21 +65)	76^b (16+60)	44^b (10+12+22)	N/A	N/A
**Sabir *et al.* (2019)**	1,116	591 (53%)	0	?	0	?	N/A	525
**Chia *et al.* (2021)**	6,982	4,588 (66%)	605	?	0	?	N/A	1,789
**Kaivola *et al.* (2022)**	3,423	2,323–2,928^c (68–86%)	≤605	407^b (66+341)	0	88	N/A	N/A
**Current sample**	4,985	0 (0%)	1,263	1,072	2,492	158	N/A	N/A

[a]These columns are populated only if an analysis was performed on a pathologically confirmed LB^- or LB^+ group. In this case the sizes of the corresponding subgroups are marked as unknown (*e.g.,* AD^+LB^+ and AD^-LB^+ if LB^+ is known). [b]The given category was subdivided into multiple phenotypes, described in **Table 3,** and a separate analysis was performed on each subgroup [28, 18, 46]. [c]The control cohort of 2,928 individuals studied in Kaivola *et al.* was part of a larger set of controls studied in Chia *et al.*, of whom 605 were pathologically confirmed [28, 13]. Therefore, at most 605 of the 2,928 controls in Kaivola *et al.* were pathologically confirmed, and, accordingly, at fewest 2,323 controls were not pathologically confirmed (only clinically evaluated).

Supplementary Table 2. Genome-wide significant loci in our study. Chr:Pos:Minor:Major is chromosome, position (genome build hg38, GRCh38), minor allele, and major allele. MAF is minor allele frequency. [OR] [CI] (*P*) is effect size reported as odds ratio with 95% confidence interval and significance. The loci besides *BIN1* and *APOE* are novel.

Variant	Chr:Pos:Minor:Major	Known locus or nearest gene	MAF	GWAS	OR [CI] (*P*)
**rs4663105**	2:127133851:C:A	*BIN1*	41.9%	AD^+LB^+ vs. AD^-LB^-	1.53 [1.35 1.75] (1.35e-10)
**rs4663105**	2:127133851:C:A	*BIN1*	41.9%	AD^+LB^- vs. AD^-LB^-	1.40 [1.26 1.56] (6.51e-10)
**rs112017605**	10:62580294:A:G	*AC024598.1/ AC067752.1*	6.0%	AD^-LB^+ vs. AD^-LB^-	3.01 [2.03 4.06] (4.60e-10)
**rs116691607**	17:30277568:T:C	*BLMH*	2.4%	AD^-LB^+ vs. AD^-LB^-	5.17 [2.88 9.29] (3.96e-08)
**rs429358**	19:44908684:C:T	*APOE*	27.1%	AD^+LB^+ vs. AD^-LB^-	4.24 [3.52 5.10] (1.49e-52)
**rs429358**	19:44908684:C:T	*APOE*	27.1%	AD^+LB^- vs. AD^-LB^-	4.22 [3.60 4.96] (1.41e-69)

Supplementary Table 3. Association of *APOE-ε*2 (rs7412) with different pathology contrasts. The first column corresponds to the current study, while the following two columns correspond to results obtained using the current sample using literature criteria to classify participants into pathology groups (**Fig. 1a–b**) [52, 28]. The last column corresponds to a result reported in the literature [48]. Effect sizes are reported as OR with 95% confidence interval [CI] and significance (*P*-value).

	**Current sample**	**Initial sample × Tsuang *et al.* (2013) criteria**	**Initial sample × Kaivola *et al.* (2022) criteria**	**Reported by Sabir *et al.* (2019)**
**AD^+LB^+ vs. AD^-LB^-**	0.39 [0.29 0.51] (3.89e-11)	0.39 [0.29 0.54] (3.90e-09)	0.38 [0.27 0.53] (9.24e-09)	N/A
**AD^+LB^- vs. AD^-LB^-**	0.42 [0.34 0.51] (2.68e-16)	0.45 [0.37 0.56] (4.52e-14)	0.44 [0.36 0.53] (9.20e-16)	N/A
**AD^-LB^+ vs. AD^-LB^-**	1.17 [0.79 1.74] (4.30e-01)	0.90 [0.65 1.24] (5.18e-01)	1.12 [0.75 1.68] (5.72e-01)	N/A
**AD^+LB^+ vs. AD^+LB^-**	0.90 [0.69 1.18] (4.55e-01)	0.84 [0.62 1.13] (2.49e-01)	0.85 [0.63 1.14] (2.78e-01)	N/A
**LB^+ vs. AD^-LB^-**	N/A	N/A	N/A	0.39 [0.26 0.59] (6.88e-06)

Supplementary Table 4. Association of rs4663105 on the *BIN1* locus and *APOE-ε*4 (rs429358) with different pathology contrasts. First two columns correspond to the current study (based on a meta-analysis) and are compared to the association in the subsets of NACC and Rush individuals separately.

	**Current sample**		**Current sample NACC individuals**		**Current sample Rush individuals**	
	**rs4663105**	**rs429358**	**rs4663105**	**rs429358**	**rs4663105**	**rs429358**
**AD^+LB^+ vs. AD^-LB^-**	1.53 [1.35 1.75] (1.35e-10)	4.24 [3.52 5.10] (1.49e-52)	1.68 [1.44 1.96] (7.01e-11)	4.54 [3.66 5.63] (4.95e-43)	1.24 [0.98 1.57] (7.88e-02)	3.48 [2.42 5.01] (1.83e-11)
**AD^+LB^- vs. AD^-LB^-**	1.40 [1.26 1.56] (6.51e-10)	4.22 [3.60 4.96] (1.41e-69)	1.52 [1.33 1.73] (3.96e-10)	4.54 [3.66 5.63] (4.95e-43)	1.19 [0.99 1.43] (6.43e-02)	3.53 [2.60 4.77] (3.56e-16)
**AD^-LB^+ vs. AD^-LB^-**	1.10 [0.85 1.41] (4.76e-01)	0.93 [0.60 1.43] (7.34e-01)	1.01 [0.72 1.42] (9.57e-01)	1.05 [0.62 1.78] (8.53e-01)	1.20 [0.83 1.72] (3.27e-01)	0.72 [0.34 1.53] (3.94e-01)
**AD^+LB^+ vs. AD^+LB^-**	1.13 [1.02 1.25] (1.91e-02)	1.01 [0.90 1.13] (8.33e-01)	1.15 [1.03 1.30] (1.38e-02)	1.01 [0.90 1.14] (8.36e-01)	1.04 [0.82 1.31] (7.72e-01)	1.01 [0.74 1.38] (9.60e-01)

Supplementary Table 5. Associations of known Alzheimer's disease risk loci reported in Bellenguez *et al.* (2022) and known Parkinson's disease risk loci reported in Chang *et al.* (2017) and Nalls *et al.* (2019) with different pathology contrasts [3, 12, 40]. Loci for which the reported variant was missing from our summary statistics are not included. Chr:Pos:Minor:Major is chromosome, position, and minor allele and major allele in our study. Lit. MAF is the frequency reported in the literature of the minor allele in our study. Lit. effect is the effect size reported in the literature. N+R MAF is the minor allele frequency in our study. Nominally significant associations ($P < 0.05$) are colored red (minor allele increases risk) and blue (minor allele decreases risk), associations with $P < 0.10$ are colored green regardless of the direction of effect.

Variant	Chr:Pos:Minor:Major	Locus	Lit. MAF	Lit. effect	Study	N+R MAF	AD⁺LB⁺ vs. AD⁻LB⁻	AD⁺LB⁻ vs. AD⁻LB⁻	AD⁻LB⁺ vs. AD⁻LB⁻	AD⁺LB⁺ vs. AD⁺LB⁻
**rs35749011**	1:155162560:A:G	*GBA*	2.4%	1.72 [1.59 1.89] (2.59e-35)	Chang *et al.* (2017)	1.3%	1.60 [0.87 2.91] (1.28e-01)	0.82 [0.47 1.44] (4.89e-01)	2.10 [0.74 5.93] (1.62e-01)	1.94 [1.24 3.06] (4.04e-03)
**rs6658353**	1:161499264:C:G	*FCGR2A*	50.1%	1.07 [1.05 1.09] (6.10e-12)	Nalls *et al.* (2019)	49.5%	0.94 [0.82 1.07] (3.53e-01)	0.97 [0.87 1.08] (5.51e-01)	0.89 [0.68 1.15] (3.64e-01)	0.99 [0.89 1.10] (8.32e-01)
**rs11578699**	1:171750629:T:C	*VAMP4*	19.5%	0.93 [0.91 0.95] (4.47e-09)	Nalls *et al.* (2019)	19.3%	1.04 [0.88 1.22] (6.48e-01)	0.98 [0.86 1.11] (7.18e-01)	1.20 [0.90 1.60] (2.22e-01)	1.06 [0.93 1.20] (4.05e-01)
**rs823118**	1:205754444:C:T	*NUCKS1/ SLC41A1*	46.7%	0.89 [0.87 0.91] (1.12e-23)	Chang *et al.* (2017)	44.4%	0.94 [0.83 1.07] (3.34e-01)	0.94 [0.85 1.05] (2.63e-01)	1.05 [0.82 1.34] (7.03e-01)	0.97 [0.88 1.08] (6.04e-01)
**rs679515**	1:207577223:T:C	*CR1*	18.8%	1.13 [1.11 1.15] (7.20e-46)	Bellenguez *et al.* (2022)	20.2%	1.09 [0.93 1.29] (2.98e-01)	1.19 [1.04 1.36] (9.99e-03)	0.91 [0.66 1.25] (5.55e-01)	0.95 [0.83 1.08] (4.22e-01)
**rs4653767**	1:226728377:C:T	*ITPKB*	31.5%	0.92 [0.90 0.94] (1.63e-11)	Chang *et al.* (2017)	29.2%	0.92 [0.80 1.06] (2.43e-01)	0.94 [0.84 1.06] (3.23e-01)	0.85 [0.65 1.12] (2.47e-01)	0.95 [0.85 1.06] (3.38e-01)
**rs10797576**	1:232528865:T:C	*SIPA1L2*	13.7%	1.12 [1.09 1.15] (8.41e-13)	Chang *et al.* (2017)	13.2%	1.04 [0.87 1.26] (6.39e-01)	1.05 [0.90 1.22] (5.34e-01)	1.18 [0.84 1.65] (3.46e-01)	1.00 [0.86 1.17] (9.63e-01)
**rs72777026**	2:9558882:G:A	*ADAM17*	14.4%	1.06 [1.04 1.08] (2.70e-08)	Bellenguez *et al.* (2022)	14.2%	1.30 [1.09 1.56] (3.92e-03)	1.17 [1.01 1.37] (4.19e-02)	1.44 [1.04 2.00] (2.81e-02)	1.13 [0.98 1.31] (8.13e-02)
**rs76116224**	2:17966582:T:A	*KCNS3*	9.6%	1.12 [1.08 1.16] (1.27e-08)	Nalls *et al.* (2019)	9.7%	0.95 [0.77 1.18] (6.57e-01)	0.94 [0.79 1.12] (4.83e-01)	1.05 [0.71 1.55] (8.03e-01)	1.06 [0.89 1.27] (5.14e-01)
**rs17020490**	2:37304796:C:T	*PRKD3*	14.5%	1.06 [1.04 1.08] (3.30e-09)	Bellenguez *et al.* (2022)	14.6%	1.10 [0.91 1.31] (3.26e-01)	1.04 [0.90 1.21] (5.81e-01)	1.07 [0.74 1.54] (7.25e-01)	1.04 [0.90 1.19] (6.19e-01)
**rs2042477**	2:95335195:A:T	*KCNIP3*	24.2%	0.94 [0.92 0.96] (1.38e-08)	Nalls *et al.* (2019)	24.7%	0.95 [0.82 1.11] (5.33e-01)	0.90 [0.79 1.01] (8.34e-02)	1.00 [0.76 1.32] (9.88e-01)	1.06 [0.94 1.20] (3.56e-01)
**rs34043159**	2:101796654:C:T	*IL1R2*	35.2%	1.08 [1.06 1.10] (5.48e-11)	Chang *et al.* (2017)	33.1%	1.00 [0.87 1.15] (9.66e-01)	0.96 [0.86 1.08] (5.06e-01)	1.08 [0.83 1.41] (5.59e-01)	1.04 [0.93 1.16] (5.30e-01)
**rs6733839**	2:127135234:T:C	*BIN1*	38.9%	1.17 [1.16 1.19] (6.10e-118)	Bellenguez *et al.* (2022)	41.9%	1.52 [1.34 1.74] (4.60e-10)	1.43 [1.29 1.60] (7.49e-11)	1.12 [0.87 1.45] (3.72e-01)	1.10 [0.99 1.22] (6.57e-02)
**rs6430538**	2:134782397:T:C	*TMEM163/ CCNT2*	48.8%	0.89 [0.87 0.91] (8.24e-24)	Chang *et al.* (2017)	43.7%	1.01 [0.89 1.15] (8.36e-01)	1.12 [1.00 1.24] (4.01e-02)	0.86 [0.67 1.10] (2.27e-01)	0.91 [0.82 1.01] (8.31e-02)

**rs353116**	2:165277122:T:C	*SCN3A*	38.5%	0.94 [0.92 0.96] (2.98e-08)	Chang *et al.* (2017)	38.7%	0.85 [0.74 0.96] (1.14e-02)	0.94 [0.84 1.04] (2.46e-01)	0.90 [0.70 1.15] (4.02e-01)	0.95 [0.86 1.06] (3.52e-01)
**rs1474055**	2:168253884:T:C	*STK39*	11.9%	1.20 [1.16 1.25] (5.68e-26)	Chang *et al.* (2017)	12.2%	0.85 [0.70 1.04] (1.17e-01)	1.00 [0.86 1.17] (9.74e-01)	0.96 [0.66 1.39] (8.16e-01)	0.88 [0.75 1.03] (1.11e-01)
**rs139643391**	2:202878716:T:TC	*WDR12*	13.1%	0.94 [0.92 0.96] (1.10e-08)	Bellenguez *et al.* (2022)	12.2%	0.97 [0.80 1.16] (7.11e-01)	0.94 [0.80 1.10] (4.33e-01)	1.15 [0.83 1.61] (4.04e-01)	0.98 [0.84 1.15] (8.22e-01)
**rs10933431**	2:233117202:G:C	*INPP5D*	23.4%	0.93 [0.92 0.95] (3.60e-18)	Bellenguez *et al.* (2022)	21.8%	0.87 [0.74 1.01] (7.37e-02)	0.79 [0.70 0.90] (3.09e-04)	1.09 [0.83 1.44] (5.42e-01)	1.07 [0.94 1.22] (2.99e-01)
**rs4073221**	3:18235996:G:T	*SATB1*	13.2%	1.10 [1.06 1.13] (1.57e-08)	Chang *et al.* (2017)	12.9%	1.04 [0.86 1.26] (6.68e-01)	1.08 [0.92 1.26] (3.55e-01)	0.97 [0.67 1.39] (8.65e-01)	1.03 [0.88 1.19] (7.40e-01)
**rs6808178**	3:28664199:T:C	*LINC00693*	37.9%	1.07 [1.05 1.09] (8.09e-12)	Nalls *et al.* (2019)	37.5%	0.90 [0.79 1.03] (1.27e-01)	0.99 [0.89 1.10] (8.79e-01)	0.84 [0.65 1.07] (1.64e-01)	0.90 [0.81 1.01] (6.81e-02)
**rs12497850**	3:48711556:G:T	*NCKIPSD/ CDC71*	34.7%	0.93 [0.91 0.96] (9.16e-09)	Chang *et al.* (2017)	34.8%	1.00 [0.87 1.14] (9.60e-01)	1.04 [0.93 1.16] (4.95e-01)	1.09 [0.84 1.41] (5.30e-01)	0.98 [0.87 1.09] (6.53e-01)
**rs115185635**	3:87471707:C:G	*CHMP2B*	3.6%	1.21 [1.10 1.33] (1.22e-04)	Chang *et al.* (2017)	3.9%	0.97 [0.69 1.34] (8.33e-01)	1.03 [0.79 1.34] (8.36e-01)	0.79 [0.40 1.56] (5.06e-01)	0.92 [0.70 1.21] (5.62e-01)
**rs55961674**	3:122478045:T:C	*KPNA1*	17.2%	1.09 [1.06 1.12] (9.98e-12)	Nalls *et al.* (2019)	16.9%	0.97 [0.82 1.15] (7.47e-01)	1.04 [0.91 1.20] (5.47e-01)	0.95 [0.68 1.32] (7.59e-01)	0.97 [0.85 1.12] (6.92e-01)
**rs11707416**	3:151391177:A:T	*MED12L*	36.7%	0.94 [0.92 0.96] (1.13e-10)	Nalls *et al.* (2019)	37.3%	1.00 [0.88 1.14] (9.93e-01)	1.07 [0.96 1.19] (2.47e-01)	1.04 [0.81 1.32] (7.83e-01)	0.95 [0.86 1.06] (3.94e-01)
**rs16824536**	3:155069722:A:G	*MME*	5.4%	0.92 [0.89 0.95] (3.60e-08)	Bellenguez *et al.* (2022)	5.4%	0.80 [0.61 1.04] (9.94e-02)	0.77 [0.62 0.96] (2.09e-02)	1.22 [0.78 1.93] (3.86e-01)	1.10 [0.87 1.40] (4.12e-01)
**rs61762319**	3:155084189:G:A	*MME*	2.6%	1.16 [1.11 1.21] (2.20e-11)	Bellenguez *et al.* (2022)	2.6%	0.97 [0.64 1.48] (8.97e-01)	1.32 [0.95 1.82] (9.44e-02)	0.66 [0.26 1.70] (3.87e-01)	0.72 [0.52 1.00] (5.34e-02)
**rs1450522**	3:161359842:G:A	*SPTSSB*	32.6%	1.06 [1.04 1.09] (5.01e-10)	Nalls *et al.* (2019)	32.0%	0.92 [0.81 1.06] (2.38e-01)	0.96 [0.86 1.08] (5.25e-01)	1.03 [0.80 1.33] (8.15e-01)	0.96 [0.86 1.07] (4.19e-01)
**rs12637471**	3:183044649:A:G	*MCCC1*	21.9%	0.85 [0.82 0.87] (2.11e-30)	Chang *et al.* (2017)	20.2%	1.03 [0.88 1.20] (7.21e-01)	0.94 [0.83 1.07] (3.35e-01)	0.85 [0.63 1.15] (2.93e-01)	1.07 [0.94 1.21] (3.30e-01)
**rs34311866**	4:958159:C:T	*TMEM175/ DGKQ*	19.9%	1.23 [1.20 1.27] (1.47e-50)	Chang *et al.* (2017)	19.1%	1.07 [0.91 1.24] (4.24e-01)	0.89 [0.78 1.02] (9.56e-02)	1.33 [1.01 1.75] (4.29e-02)	1.22 [1.07 1.38] (2.83e-03)
**rs6846529**	4:11023507:C:T	*CLNK/ HS3ST1*	28.3%	1.07 [1.05 1.08] (2.20e-17)	Bellenguez *et al.* (2022)	28.0%	1.11 [0.96 1.28] (1.46e-01)	1.15 [1.02 1.29] (2.12e-02)	1.01 [0.77 1.32] (9.49e-01)	0.97 [0.86 1.08] (5.66e-01)
**rs11724635**	4:15735478:C:A	*FAM200B/ CD38*	43.7%	0.90 [0.88 0.92] (1.22e-19)	Chang *et al.* (2017)	45.3%	1.01 [0.89 1.15] (8.64e-01)	0.97 [0.87 1.08] (5.37e-01)	0.71 [0.55 0.92] (9.03e-03)	1.05 [0.95 1.17] (3.28e-01)
**rs34025766**	4:17967188:A:T	*LCORL*	15.9%	0.92 [0.90 0.94] (2.87e-10)	Nalls *et al.* (2019)	16.4%	1.15 [0.97 1.36] (1.11e-01)	1.04 [0.90 1.20] (5.81e-01)	0.92 [0.65 1.29] (6.25e-01)	1.10 [0.96 1.26] (1.84e-01)
**rs2245466**	4:40197226:G:C	*RHOH*	34.3%	1.05 [1.03 1.06] (1.20e-09)	Bellenguez *et al.* (2022)	33.2%	1.06 [0.93 1.22] (3.74e-01)	1.04 [0.93 1.17] (5.05e-01)	0.97 [0.74 1.27] (8.27e-01)	1.04 [0.93 1.16] (5.13e-01)
**rs6812193**	4:76277833:T:C	*FAM47E*	39.8%	0.92 [0.90 0.94] (1.43e-14)	Chang *et al.* (2017)	37.0%	0.91 [0.80 1.03] (1.44e-01)	0.98 [0.88 1.09] (7.55e-01)	0.92 [0.72 1.18] (5.00e-01)	0.95 [0.85 1.05] (3.06e-01)

**rs356182**	4:89704960:G:A	*SNCA*	37.5%	0.75 [0.74 0.77] (5.21e-123)	Chang *et al.* (2017)	34.9%	0.90 [0.79 1.03] (1.37e-01)	0.95 [0.85 1.07] (4.04e-01)	0.74 [0.57 0.97] (2.60e-02)	0.97 [0.87 1.08] (5.38e-01)
**rs78738012**	4:113439216:C:T	*ANK2/ CAMK2D*	10.6%	1.13 [1.09 1.17] (4.78e-11)	Chang *et al.* (2017)	10.0%	1.05 [0.86 1.30] (6.18e-01)	1.00 [0.84 1.19] (9.78e-01)	1.07 [0.72 1.61] (7.27e-01)	1.02 [0.86 1.21] (8.27e-01)
**rs62333164**	4:169662006:A:G	*CLCN3*	32.6%	0.94 [0.92 0.96] (2.00e-10)	Nalls *et al.* (2019)	32.7%	1.06 [0.93 1.21] (3.73e-01)	1.14 [1.02 1.28] (1.74e-02)	1.14 [0.88 1.46] (3.25e-01)	0.96 [0.86 1.07] (4.79e-01)
**rs112403360**	5:14724304:A:T	*ANKH*	7.3%	1.09 [1.06 1.12] (2.30e-09)	Bellenguez *et al.* (2022)	7.4%	1.05 [0.81 1.36] (7.20e-01)	1.30 [1.06 1.59] (1.13e-02)	1.24 [0.80 1.93] (3.37e-01)	0.82 [0.67 1.00] (5.05e-02)
**rs2694528**	5:60978096:C:A	*ELOVL7*	11.5%	1.15 [1.11 1.20] (4.84e-15)	Chang *et al.* (2017)	8.5%	0.89 [0.71 1.12] (3.13e-01)	0.93 [0.78 1.12] (4.41e-01)	0.76 [0.47 1.23] (2.69e-01)	1.01 [0.83 1.22] (9.46e-01)
**rs62374257**	5:86927378:C:T	*COX7C*	23.0%	1.07 [1.05 1.09] (1.40e-15)	Bellenguez *et al.* (2022)	22.8%	1.21 [1.04 1.41] (1.63e-02)	1.26 [1.11 1.43] (3.83e-04)	1.17 [0.87 1.57] (3.09e-01)	0.96 [0.85 1.08] (5.03e-01)
**rs26431**	5:103030090:G:C	*PAM*	29.7%	0.94 [0.92 0.96] (1.57e-09)	Nalls *et al.* (2019)	30.6%	1.01 [0.88 1.16] (9.07e-01)	1.00 [0.89 1.12] (9.32e-01)	1.09 [0.83 1.42] (5.41e-01)	1.05 [0.94 1.18] (3.70e-01)
**rs11950533**	5:134863415:A:C	*C5orf24*	10.2%	0.91 [0.88 0.94] (7.16e-09)	Nalls *et al.* (2019)	10.4%	0.84 [0.68 1.02] (8.48e-02)	0.83 [0.70 0.98] (2.70e-02)	0.72 [0.47 1.10] (1.33e-01)	0.96 [0.81 1.14] (6.48e-01)
**rs871269**	5:151052827:T:C	*TNIP1*	32.6%	0.96 [0.95 0.97] (8.70e-09)	Bellenguez *et al.* (2022)	32.4%	0.95 [0.83 1.09] (4.59e-01)	0.95 [0.85 1.06] (3.78e-01)	0.99 [0.77 1.29] (9.67e-01)	1.03 [0.93 1.15] (5.70e-01)
**rs113706587**	5:180201150:A:G	*RASGEF1C*	11.0%	1.09 [1.07 1.12] (2.20e-16)	Bellenguez *et al.* (2022)	10.8%	1.09 [0.89 1.34] (4.03e-01)	1.12 [0.95 1.33] (1.85e-01)	0.92 [0.61 1.39] (6.95e-01)	0.96 [0.81 1.13] (6.00e-01)
**rs9468199**	6:27713436:A:G	*ZNF184*	17.2%	1.11 [1.08 1.14] (1.46e-12)	Chang *et al.* (2017)	17.8%	1.07 [0.91 1.27] (4.16e-01)	1.00 [0.87 1.14] (9.80e-01)	1.02 [0.74 1.41] (9.14e-01)	1.06 [0.93 1.21] (3.77e-01)
**rs9261484**	6:30140906:T:C	*TRIM40*	24.5%	0.94 [0.92 0.96] (1.62e-08)	Nalls *et al.* (2019)	25.1%	1.08 [0.94 1.25] (2.75e-01)	1.06 [0.94 1.19] (3.46e-01)	1.01 [0.77 1.33] (9.40e-01)	1.01 [0.90 1.14] (8.47e-01)
**rs6605556**	6:32615322:G:A	*HLA*	16.1%	0.91 [0.90 0.93] (7.10e-20)	Bellenguez *et al.* (2022)	16.0%	0.81 [0.69 0.96] (1.67e-02)	0.87 [0.76 1.00] (4.57e-02)	1.01 [0.74 1.38] (9.43e-01)	0.97 [0.84 1.12] (6.81e-01)
**rs9275326**	6:32698883:T:C	*HLA-DRB6/ HLA-DQA1*	11.4%	0.85 [0.82 0.89] (1.26e-13)	Chang *et al.* (2017)	9.8%	0.74 [0.60 0.91] (4.75e-03)	0.83 [0.70 0.98] (2.84e-02)	0.97 [0.66 1.44] (8.86e-01)	0.88 [0.74 1.06] (1.74e-01)
**rs10947943**	6:41036354:A:G	*TREM2*	14.2%	0.94 [0.93 0.96] (1.10e-09)	Bellenguez *et al.* (2022)	14.4%	0.89 [0.75 1.07] (2.06e-01)	0.86 [0.75 1.00] (4.51e-02)	1.05 [0.76 1.46] (7.71e-01)	1.06 [0.91 1.22] (4.51e-01)
**rs143332484**	6:41161469:T:C	*TREM2*	1.3%	1.41 [1.32 1.50] (2.80e-25)	Bellenguez *et al.* (2022)	1.3%	1.99 [1.09 3.62] (2.50e-02)	1.87 [1.11 3.16] (1.97e-02)	1.69 [0.55 5.18] (3.60e-01)	1.35 [0.89 2.04] (1.58e-01)
**rs7767350**	6:47517390:T:C	*CD2AP*	27.1%	1.08 [1.06 1.09] (7.90e-22)	Bellenguez *et al.* (2022)	28.2%	1.12 [0.97 1.29] (1.18e-01)	1.12 [0.99 1.25] (6.63e-02)	0.98 [0.74 1.28] (8.69e-01)	0.99 [0.88 1.11] (8.49e-01)
**rs12528068**	6:71778059:T:C	*RIMS1*	28.4%	1.07 [1.05 1.09] (1.63e-10)	Nalls *et al.* (2019)	27.9%	1.02 [0.89 1.18] (7.55e-01)	1.10 [0.98 1.24] (9.40e-02)	0.94 [0.72 1.24] (6.83e-01)	0.96 [0.85 1.07] (4.36e-01)
**rs997368**	6:111922088:G:A	*FYN*	19.5%	0.93 [0.91 0.95] (1.84e-09)	Nalls *et al.* (2019)	19.6%	0.92 [0.78 1.08] (3.11e-01)	0.92 [0.78 1.08] (3.11e-01)	0.92 [0.78 1.08] (3.11e-01)	0.88 [0.77 1.01] (6.21e-02)
**rs785129**	6:114291731:T:C	*HS3ST5*	35.0%	1.04 [1.03 1.06] (2.40e-09)	Bellenguez *et al.* (2022)	34.7%	1.19 [1.04 1.36] (1.25e-02)	1.11 [0.99 1.24] (7.66e-02)	1.13 [0.87 1.47] (3.41e-01)	1.05 [0.94 1.17] (4.25e-01)
**rs75859381**	6:132889222:C:T	*RPS12*	3.3%	1.25 [1.16 1.33] (1.04e-10)	Nalls *et al.* (2019)	3.1%	0.83 [0.58 1.19] (3.07e-01)	0.80 [0.60 1.08] (1.48e-01)	1.08 [0.54 2.20] (8.21e-01)	1.13 [0.85 1.52] (3.99e-01)

**rs6943429**	7:7817263:T:C	*UMAD1*	42.0%	1.05 [1.03 1.06] (1.00e-10)	Bellenguez *et al.* (2022)	42.6%	1.00 [0.88 1.15] (9.65e-01)	1.08 [0.97 1.21] (1.44e-01)	0.83 [0.65 1.07] (1.52e-01)	0.91 [0.82 1.02] (9.94e-02)
**rs10952097**	7:8204382:T:C	*ICA1*	11.4%	1.07 [1.05 1.10] (6.80e-09)	Bellenguez *et al.* (2022)	10.0%	1.08 [0.87 1.34] (4.67e-01)	1.10 [0.93 1.31] (2.73e-01)	0.80 [0.51 1.24] (3.09e-01)	1.01 [0.85 1.20] (9.14e-01)
**rs13237518**	7:12229967:A:C	*TMEM106B*	41.1%	0.96 [0.94 0.97] (4.90e-11)	Bellenguez *et al.* (2022)	42.1%	1.23 [1.08 1.39] (1.29e-03)	1.12 [1.01 1.24] (3.34e-02)	1.22 [0.97 1.55] (9.14e-02)	1.05 [0.95 1.17] (3.09e-01)
**rs199347**	7:23254127:G:A	*KLHL7/ NUPL2/ GPNMB*	36.8%	0.91 [0.89 0.93] (3.51e-18)	Chang *et al.* (2017)	41.5%	1.08 [0.95 1.23] (2.39e-01)	1.13 [1.01 1.25] (2.57e-02)	1.04 [0.81 1.32] (7.74e-01)	0.92 [0.83 1.03] (1.40e-01)
**rs1160871**	7:28129126:G:GTCTT	*JAZF1*	22.2%	0.95 [0.93 0.97] (9.80e-09)	Bellenguez *et al.* (2022)	21.9%	0.92 [0.79 1.07] (2.70e-01)	0.97 [0.86 1.10] (6.46e-01)	1.07 [0.80 1.42] (6.47e-01)	0.98 [0.86 1.11] (7.24e-01)
**rs6966331**	7:37844191:T:C	*NME8*	34.9%	0.96 [0.94 0.97] (4.60e-10)	Bellenguez *et al.* (2022)	33.5%	1.03 [0.90 1.18] (6.75e-01)	0.91 [0.82 1.02] (1.11e-01)	0.98 [0.76 1.25] (8.56e-01)	1.11 [0.99 1.24] (7.40e-02)
**rs76928645**	7:54873635:T:C	*SEC61G*	10.3%	0.93 [0.91 0.95] (1.60e-10)	Bellenguez *et al.* (2022)	10.2%	0.81 [0.65 1.00] (4.84e-02)	0.90 [0.76 1.07] (2.33e-01)	1.10 [0.76 1.59] (6.02e-01)	0.92 [0.77 1.10] (3.59e-01)
**rs76949143**	7:66544864:A:T	*GS1-124K5.11*	5.1%	0.87 [0.82 0.91] (1.43e-08)	Nalls *et al.* (2019)	4.7%	1.09 [0.81 1.45] (5.77e-01)	0.98 [0.77 1.25] (8.99e-01)	0.90 [0.49 1.64] (7.34e-01)	1.08 [0.85 1.37] (5.32e-01)
**rs7384878**	7:100334426:C:T	*ZCWPW1/ NYAP1*	31.0%	0.92 [0.91 0.94] (1.10e-26)	Bellenguez *et al.* (2022)	29.3%	0.95 [0.82 1.09] (4.43e-01)	0.85 [0.75 0.95] (5.45e-03)	1.26 [0.97 1.63] (8.23e-02)	0.94 [0.84 1.05] (2.67e-01)
**rs11771145**	7:143413669:A:G	*EPHA1*	34.8%	0.95 [0.93 0.96] (3.30e-14)	Bellenguez *et al.* (2022)	34.0%	0.95 [0.84 1.09] (4.84e-01)	0.92 [0.82 1.02] (1.26e-01)	1.23 [0.96 1.57] (1.01e-01)	1.02 [0.92 1.14] (7.14e-01)
**rs1065712**	8:11844613:C:G	*CTSB*	5.3%	1.09 [1.06 1.12] (1.90e-09)	Bellenguez *et al.* (2022)	5.8%	1.34 [1.03 1.75] (3.22e-02)	1.09 [0.87 1.38] (4.46e-01)	0.85 [0.48 1.49] (5.67e-01)	1.23 [1.00 1.52] (5.43e-02)
**rs2740594**	8:11849665:G:A	*CTSB*	24.7%	0.92 [0.89 0.93] (5.91e-12)	Chang *et al.* (2017)	27.0%	1.11 [0.96 1.28] (1.47e-01)	1.05 [0.93 1.18] (4.01e-01)	0.93 [0.71 1.23] (6.08e-01)	1.01 [0.90 1.13] (8.84e-01)
**rs591323**	8:16839582:A:G	*MICU3*	29.3%	0.91 [0.89 0.94] (2.38e-11)	Chang *et al.* (2017)	27.2%	1.18 [1.02 1.36] (2.28e-02)	1.04 [0.92 1.17] (5.58e-01)	1.11 [0.85 1.45] (4.27e-01)	1.08 [0.97 1.22] (1.69e-01)
**rs2280104**	8:22668467:T:C	*SORBS3/ PDLIM2/ C8orf58/ BIN3*	36.7%	1.07 [1.04 1.09] (2.53e-08)	Chang *et al.* (2017)	35.3%	0.97 [0.85 1.12] (6.98e-01)	1.03 [0.92 1.15] (6.12e-01)	1.10 [0.85 1.41] (4.78e-01)	0.95 [0.85 1.06] (3.43e-01)
**rs73223431**	8:27362470:T:C	*PTK2B*	36.9%	1.07 [1.06 1.08] (4.00e-22)	Bellenguez *et al.* (2022)	36.4%	1.13 [0.99 1.29] (7.98e-02)	1.16 [1.04 1.29] (6.67e-03)	1.23 [0.96 1.58] (1.04e-01)	1.00 [0.90 1.11] (9.94e-01)
**rs11787077**	8:27607795:T:C	*CLU*	39.2%	0.91 [0.90 0.92] (1.70e-44)	Bellenguez *et al.* (2022)	38.5%	0.85 [0.74 0.97] (1.33e-02)	0.89 [0.80 0.99] (3.43e-02)	0.98 [0.77 1.26] (8.81e-01)	0.95 [0.85 1.06] (3.62e-01)
**rs2086641**	8:129889663:C:T	*FAM49B*	27.7%	1.06 [1.04 1.09] (1.81e-08)	Nalls *et al.* (2019)	27.5%	0.97 [0.84 1.12] (6.69e-01)	1.00 [0.89 1.13] (9.38e-01)	1.23 [0.94 1.62] (1.32e-01)	1.03 [0.92 1.16] (5.74e-01)
**rs34173062**	8:144103704:A:G	*SHARPIN*	8.1%	1.13 [1.09 1.16] (1.70e-16)	Bellenguez *et al.* (2022)	8.3%	0.95 [0.75 1.20] (6.48e-01)	1.12 [0.93 1.35] (2.34e-01)	1.31 [0.89 1.95] (1.76e-01)	0.87 [0.72 1.06] (1.66e-01)
**rs13294100**	9:17579692:T:G	*SH3GL2*	37.1%	0.92 [0.89 0.94] (4.84e-13)	Chang *et al.* (2017)	35.5%	1.00 [0.87 1.14] (9.93e-01)	0.94 [0.84 1.05] (2.74e-01)	0.93 [0.72 1.20] (5.82e-01)	1.04 [0.93 1.15] (5.14e-01)
**rs6476434**	9:34046393:C:T	*UBAP2*	26.6%	1.06 [1.04 1.09] (6.58e-09)	Nalls *et al.* (2019)	27.1%	1.02 [0.88 1.18] (7.94e-01)	0.99 [0.88 1.11] (8.28e-01)	0.88 [0.66 1.16] (3.51e-01)	1.05 [0.93 1.18] (4.15e-01)

**rs1800978**	9:104903697:G:C	*ABCA1*	13.0%	1.06 [1.04 1.08] (1.60e-09)	Bellenguez *et al.* (2022)	13.8%	1.02 [0.85 1.22] (8.18e-01)	0.95 [0.81 1.10] (4.68e-01)	0.74 [0.50 1.09] (1.28e-01)	1.09 [0.95 1.27] (2.23e-01)
**rs7912495**	10:11676714:G:A	*ECHDC3*	46.2%	1.06 [1.05 1.08] (9.70e-19)	Bellenguez *et al.* (2022)	46.1%	1.17 [1.03 1.33] (1.47e-02)	1.13 [1.01 1.25] (2.56e-02)	1.24 [0.97 1.59] (8.33e-02)	1.04 [0.94 1.15] (4.86e-01)
**rs10906923**	10:15527599:C:A	*FAM171A1*	30.6%	0.93 [0.91 0.96] (1.35e-08)	Chang *et al.* (2017)	32.4%	1.03 [0.90 1.18] (6.87e-01)	0.99 [0.89 1.11] (8.71e-01)	0.83 [0.64 1.08] (1.68e-01)	1.05 [0.94 1.17] (4.23e-01)
**rs7068231**	10:60025170:T:G	*ANK3*	40.3%	0.95 [0.94 0.96] (3.30e-13)	Bellenguez *et al.* (2022)	39.5%	1.02 [0.89 1.17] (7.55e-01)	1.01 [0.91 1.13] (8.30e-01)	0.87 [0.68 1.12] (2.77e-01)	0.96 [0.86 1.07] (4.59e-01)
**rs6586028**	10:80494228:C:T	*TSPAN14*	19.6%	0.93 [0.91 0.94] (2.00e-19)	Bellenguez *et al.* (2022)	19.4%	0.90 [0.76 1.05] (1.85e-01)	0.93 [0.81 1.06] (2.73e-01)	1.08 [0.81 1.44] (6.02e-01)	0.98 [0.86 1.12] (7.90e-01)
**rs6584063**	10:96266650:G:A	*BLNK*	4.3%	0.89 [0.86 0.92] (6.70e-11)	Bellenguez *et al.* (2022)	3.5%	0.83 [0.60 1.15] (2.63e-01)	0.80 [0.61 1.04] (9.85e-02)	0.85 [0.46 1.58] (6.07e-01)	1.07 [0.80 1.44] (6.51e-01)
**rs10748818**	10:102255522:G:A	*GBF1*	14.9%	1.09 [1.05 1.11] (1.05e-09)	Nalls *et al.* (2019)	14.5%	0.90 [0.75 1.08] (2.70e-01)	0.98 [0.85 1.13] (7.65e-01)	0.94 [0.66 1.33] (7.12e-01)	0.91 [0.79 1.06] (2.28e-01)
**rs117896735**	10:119776815:A:G	*BAG3*	1.2%	1.65 [1.48 1.85] (2.23e-19)	Chang *et al.* (2017)	1.7%	1.62 [0.98 2.67] (5.93e-02)	1.37 [0.89 2.12] (1.56e-01)	1.03 [0.35 3.04] (9.56e-01)	1.10 [0.75 1.60] (6.28e-01)
**rs7908662**	10:122413396:G:A	*PLEKHA1*	46.7%	0.96 [0.95 0.97] (2.60e-09)	Bellenguez *et al.* (2022)	47.4%	1.00 [0.89 1.14] (9.41e-01)	0.93 [0.83 1.03] (1.55e-01)	0.95 [0.75 1.21] (6.73e-01)	1.05 [0.95 1.17] (3.26e-01)
**rs7938782**	11:10537230:G:A	*RNF141*	12.2%	0.92 [0.89 0.94] (2.12e-09)	Nalls *et al.* (2019)	12.6%	1.05 [0.87 1.27] (5.97e-01)	0.98 [0.83 1.14] (7.55e-01)	1.19 [0.85 1.68] (3.13e-01)	1.09 [0.94 1.28] (2.54e-01)
**rs10437655**	11:47370397:A:G	*CELF1/ SPI1*	39.9%	1.06 [1.04 1.07] (5.30e-14)	Bellenguez *et al.* (2022)	39.8%	1.12 [0.98 1.27] (8.43e-02)	1.07 [0.96 1.18] (2.29e-01)	1.01 [0.79 1.30] (9.17e-01)	1.04 [0.93 1.15] (5.05e-01)
**rs1582763**	11:60254475:A:G	*MS4A*	37.1%	0.91 [0.90 0.92] (3.70e-42)	Bellenguez *et al.* (2022)	36.2%	0.97 [0.86 1.11] (7.01e-01)	0.97 [0.87 1.08] (5.32e-01)	1.03 [0.80 1.31] (8.28e-01)	1.05 [0.94 1.17] (3.64e-01)
**rs3793947**	11:83833429:A:G	*DLG2*	46.3%	0.93 [0.91 0.95] (3.72e-09)	Chang *et al.* (2017)	43.7%	0.96 [0.84 1.09] (5.07e-01)	1.01 [0.91 1.12] (9.24e-01)	1.06 [0.83 1.34] (6.62e-01)	0.92 [0.83 1.02] (1.33e-01)
**rs3851179**	11:86157598:T:C	*PICALM*	35.8%	0.90 [0.89 0.92] (3.00e-48)	Bellenguez *et al.* (2022)	35.2%	0.91 [0.79 1.04] (1.51e-01)	0.86 [0.77 0.96] (7.92e-03)	1.05 [0.82 1.36] (6.99e-01)	1.06 [0.95 1.18] (3.11e-01)
**rs74685827**	11:121482368:G:T	*SORL1*	1.9%	1.19 [1.13 1.25] (2.80e-11)	Bellenguez *et al.* (2022)	2.1%	1.03 [0.65 1.64] (9.08e-01)	1.14 [0.78 1.67] (5.08e-01)	1.84 [0.88 3.86] (1.06e-01)	0.90 [0.63 1.28] (5.45e-01)
**rs11218343**	11:121564878:C:T	*SORL1*	3.9%	0.84 [0.81 0.87] (1.40e-21)	Bellenguez *et al.* (2022)	3.6%	1.02 [0.74 1.39] (9.15e-01)	0.72 [0.55 0.95] (2.12e-02)	1.72 [1.02 2.90] (4.36e-02)	1.32 [1.00 1.73] (4.79e-02)
**rs329648**	11:133895472:T:C	*MIR4697*	32.7%	1.09 [1.07 1.12] (1.11e-13)	Chang *et al.* (2017)	35.0%	0.96 [0.84 1.10] (5.66e-01)	1.10 [0.98 1.23] (1.11e-01)	1.03 [0.79 1.33] (8.33e-01)	0.88 [0.79 0.99] (2.62e-02)
**rs76904798**	12:40220632:T:C	*LRRK2*	13.2%	1.15 [1.12 1.19] (1.21e-19)	Chang *et al.* (2017)	13.0%	0.90 [0.75 1.08] (2.66e-01)	0.89 [0.77 1.04] (1.38e-01)	0.85 [0.59 1.22] (3.68e-01)	0.98 [0.84 1.14] (8.10e-01)
**rs7134559**	12:46025303:T:C	*SCAF11*	40.4%	0.95 [0.93 0.97] (3.96e-08)	Nalls *et al.* (2019)	39.8%	0.92 [0.81 1.05] (2.04e-01)	0.93 [0.84 1.04] (2.05e-01)	1.00 [0.79 1.27] (1.00e+00)	0.98 [0.88 1.09] (7.35e-01)
**rs6489896**	12:113281983:C:T	*TPCN1*	7.6%	1.08 [1.05 1.10] (1.80e-09)	Bellenguez *et al.* (2022)	7.0%	1.31 [1.01 1.69] (4.04e-02)	1.22 [0.99 1.52] (6.46e-02)	1.29 [0.80 2.10] (3.00e-01)	1.00 [0.82 1.22] (9.80e-01)
**rs11060180**	12:122819039:G:A	*OGFOD2*	45.0%	0.90 [0.88 0.92] (2.05e-20)	Chang *et al.* (2017)	45.3%	1.06 [0.94 1.20] (3.58e-01)	1.06 [0.95 1.17] (2.83e-01)	1.10 [0.87 1.40] (4.16e-01)	0.97 [0.87 1.07] (5.40e-01)

**rs11610045**	12:132487182:A:G	*FBRSL1*	49.0%	1.06 [1.04 1.08] (1.77e-10)	Nalls *et al.* (2019)	48.2%	0.86 [0.76 0.98] (2.03e-02)	0.91 [0.82 1.01] (7.42e-02)	0.92 [0.71 1.18] (4.92e-01)	0.97 [0.87 1.07] (5.27e-01)
**rs9568188**	13:49353596:C:T	*CAB39L*	26.0%	0.94 [0.92 0.96] (1.15e-08)	Nalls *et al.* (2019)	26.9%	0.97 [0.84 1.12] (6.81e-01)	1.01 [0.90 1.14] (8.43e-01)	0.95 [0.73 1.25] (7.24e-01)	0.94 [0.84 1.06] (3.26e-01)
**rs4771268**	13:97212767:T:C	*MBNL2*	23.0%	1.07 [1.05 1.09] (1.45e-09)	Nalls *et al.* (2019)	22.6%	0.93 [0.79 1.08] (3.39e-01)	1.02 [0.90 1.16] (7.04e-01)	1.12 [0.84 1.48] (4.45e-01)	0.91 [0.80 1.03] (1.36e-01)
**rs12147950**	14:37520065:T:C	*MIPOL1*	43.8%	0.95 [0.93 0.97] (3.54e-08)	Nalls *et al.* (2019)	43.2%	1.07 [0.94 1.22] (3.22e-01)	1.10 [0.99 1.22] (8.29e-02)	1.29 [1.00 1.65] (4.59e-02)	0.95 [0.86 1.06] (3.49e-01)
**rs17125924**	14:52924962:G:A	*FERMT2*	8.9%	1.10 [1.07 1.12] (8.30e-16)	Bellenguez *et al.* (2022)	9.0%	1.27 [1.00 1.60] (4.95e-02)	1.38 [1.14 1.67] (8.73e-04)	1.17 [0.75 1.82] (4.79e-01)	1.17 [0.75 1.82] (4.79e-01)
**rs11158026**	14:54882151:T:C	*GCH1*	30.7%	0.91 [0.89 0.93] (4.30e-16)	Chang *et al.* (2017)	33.2%	0.95 [0.84 1.09] (4.89e-01)	0.98 [0.88 1.09] (6.80e-01)	0.94 [0.73 1.20] (6.02e-01)	0.97 [0.87 1.08] (5.98e-01)
**rs1555399**	14:67517653:A:T	*TMEM229B*	45.6%	0.92 [0.90 0.94] (9.61e-11)	Chang *et al.* (2017)	48.5%	1.11 [0.98 1.25] (1.17e-01)	1.00 [0.91 1.11] (9.41e-01)	0.91 [0.72 1.16] (4.37e-01)	1.10 [1.00 1.22] (5.79e-02)
**rs3742785**	14:74906331:C:A	*RPS6KL1*	21.3%	0.93 [0.91 0.95] (1.92e-09)	Nalls *et al.* (2019)	21.6%	1.12 [0.96 1.30] (1.55e-01)	1.10 [0.97 1.24] (1.58e-01)	1.11 [0.83 1.49] (4.67e-01)	1.02 [0.90 1.15] (7.68e-01)
**rs8005172**	14:88006268:T:C	*GALC*	42.4%	1.08 [1.05 1.10] (8.77e-11)	Chang *et al.* (2017)	43.4%	1.03 [0.91 1.17] (6.33e-01)	0.96 [0.87 1.07] (4.70e-01)	0.89 [0.70 1.14] (3.54e-01)	1.06 [0.96 1.18] (2.42e-01)
**rs7401792**	14:92464917:G:A	*SLC24A4/ RIN3*	37.1%	1.04 [1.02 1.05] (4.80e-08)	Bellenguez *et al.* (2022)	36.8%	1.03 [0.90 1.18] (6.87e-01)	1.10 [0.99 1.23] (7.90e-02)	0.92 [0.71 1.20] (5.41e-01)	0.96 [0.86 1.07] (4.44e-01)
**rs12590654**	14:92472511:A:G	*SLC24A4/ RIN3*	32.8%	0.93 [0.92 0.95] (4.20e-21)	Bellenguez *et al.* (2022)	33.2%	0.89 [0.78 1.02] (1.04e-01)	1.00 [0.89 1.11] (9.49e-01)	1.30 [1.00 1.67] (4.65e-02)	0.94 [0.85 1.05] (2.94e-01)
**rs7157106**	14:105761758:A:G	*IGH*	36.0%	1.05 [1.03 1.07] (2.00e-08)	Bellenguez *et al.* (2022)	31.7%	1.05 [0.92 1.21] (4.62e-01)	1.10 [0.98 1.24] (9.96e-02)	0.91 [0.69 1.20] (5.07e-01)	0.97 [0.87 1.09] (6.05e-01)
**rs8025980**	15:50701814:G:A	*SPPL2A*	34.5%	0.96 [0.94 0.97] (1.30e-08)	Bellenguez *et al.* (2022)	35.1%	0.99 [0.86 1.12] (8.27e-01)	0.96 [0.86 1.07] (4.46e-01)	0.96 [0.75 1.23] (7.56e-01)	1.05 [0.95 1.17] (3.54e-01)
**rs602602**	15:58764824:A:T	*ADAM10*	28.0%	0.94 [0.93 0.96] (2.10e-15)	Bellenguez *et al.* (2022)	28.0%	1.00 [0.87 1.16] (9.62e-01)	1.00 [0.90 1.13] (9.40e-01)	1.08 [0.82 1.43] (5.67e-01)	0.97 [0.87 1.09] (6.14e-01)
**rs2414739**	15:61701935:G:A	*VPS13C*	29.2%	0.91 [0.89 0.93] (3.94e-14)	Chang *et al.* (2017)	26.5%	0.98 [0.85 1.13] (7.48e-01)	0.91 [0.81 1.03] (1.36e-01)	0.87 [0.66 1.15] (3.43e-01)	1.06 [0.94 1.19] (3.58e-01)
**rs117618017**	15:63277703:T:C	*APH1B*	14.4%	1.11 [1.09 1.13] (2.20e-25)	Bellenguez *et al.* (2022)	13.6%	1.15 [0.95 1.39] (1.43e-01)	1.20 [1.03 1.40] (2.00e-02)	1.44 [1.02 2.02] (3.76e-02)	0.96 [0.83 1.12] (6.04e-01)
**rs3848143**	15:64131307:G:A	*SNX1*	22.0%	1.05 [1.04 1.07] (8.40e-11)	Bellenguez *et al.* (2022)	22.1%	1.04 [0.89 1.22] (5.97e-01)	1.05 [0.92 1.19] (5.00e-01)	0.94 [0.69 1.27] (6.68e-01)	1.00 [0.88 1.13] (9.46e-01)
**rs12592898**	15:78936857:A:G	*CTSH*	13.3%	0.94 [0.92 0.96] (4.20e-09)	Bellenguez *et al.* (2022)	12.6%	0.87 [0.72 1.06] (1.65e-01)	0.90 [0.77 1.05] (1.74e-01)	0.89 [0.63 1.28] (5.38e-01)	0.94 [0.80 1.10] (4.36e-01)
**rs11343**	16:19268142:T:G	*COQ7*	45.4%	1.07 [1.05 1.10] (9.13e-11)	Chang *et al.* (2017)	44.4%	1.06 [0.93 1.20] (3.98e-01)	0.99 [0.89 1.10] (8.64e-01)	0.94 [0.74 1.20] (6.37e-01)	1.10 [0.99 1.22] (9.05e-02)
**rs2904880**	16:28933075:C:G	*CD19*	30.9%	0.94 [0.92 0.96] (7.87e-10)	Nalls *et al.* (2019)	31.6%	1.00 [0.87 1.15] (9.70e-01)	1.08 [0.97 1.21] (1.62e-01)	1.02 [0.78 1.32] (8.95e-01)	0.95 [0.85 1.06] (3.88e-01)

**rs1140239**	16:30010081:T:C	*DOC2A*	37.9%	0.94 [0.93 0.96] (2.60e-13)	Bellenguez *et al.* (2022)	38.0%	0.81 [0.71 0.93] (3.17e-03)	0.90 [0.81 1.00] (5.45e-02)	0.79 [0.61 1.02] (6.90e-02)	0.96 [0.86 1.07] (4.73e-01)
**rs14235**	16:31110472:A:G	*ZNF646/ KAT8*	39.7%	1.08 [1.06 1.10] (5.44e-12)	Chang *et al.* (2017)	37.7%	1.01 [0.88 1.16] (8.69e-01)	1.05 [0.94 1.17] (3.90e-01)	1.08 [0.84 1.39] (5.48e-01)	0.98 [0.88 1.09] (6.70e-01)
**rs889555**	16:31111250:T:C	*KAT8*	28.1%	0.95 [0.94 0.97] (2.00e-11)	Bellenguez *et al.* (2022)	27.8%	0.93 [0.81 1.08] (3.46e-01)	0.91 [0.81 1.02] (1.12e-01)	0.89 [0.67 1.17] (4.04e-01)	1.00 [0.89 1.12] (9.71e-01)
**rs6500328**	16:50702745:G:A	*NOD2*	40.1%	0.94 [0.93 0.96] (1.82e-09)	Nalls *et al.* (2019)	41.3%	1.13 [0.99 1.28] (6.83e-02)	1.10 [0.99 1.23] (6.40e-02)	0.89 [0.69 1.14] (3.61e-01)	1.03 [0.93 1.14] (5.84e-01)
**rs4784227**	16:52565276:T:C	*TOX3*	26.5%	1.09 [1.06 1.12] (9.75e-11)	Chang *et al.* (2017)	24.7%	0.96 [0.83 1.11] (5.91e-01)	0.96 [0.85 1.08] (5.34e-01)	0.73 [0.54 0.98] (3.72e-02)	1.00 [0.89 1.13] (9.54e-01)
**rs4985556**	16:70660097:A:C	*IL34*	11.5%	1.07 [1.05 1.09] (6.00e-10)	Bellenguez *et al.* (2022)	11.5%	1.21 [0.99 1.47] (6.04e-02)	1.03 [0.87 1.22] (7.14e-01)	0.92 [0.61 1.37] (6.74e-01)	1.14 [0.97 1.33] (1.01e-01)
**rs450674**	16:79574511:C:T	*MAF*	37.3%	0.96 [0.95 0.98] (3.20e-08)	Bellenguez *et al.* (2022)	36.8%	0.96 [0.85 1.10] (5.89e-01)	0.87 [0.78 0.97] (1.01e-02)	0.96 [0.75 1.24] (7.74e-01)	1.09 [0.98 1.21] (1.29e-01)
**rs12446759**	16:81739398:G:A	*PLCG2*	40.3%	0.95 [0.94 0.96] (1.20e-13)	Bellenguez *et al.* (2022)	39.8%	1.04 [0.91 1.19] (5.44e-01)	0.96 [0.86 1.06] (4.08e-01)	1.04 [0.81 1.33] (7.74e-01)	1.07 [0.96 1.19] (2.14e-01)
**rs16941239**	16:86420604:A:T	*FOXF1*	2.9%	1.13 [1.08 1.17] (1.30e-08)	Bellenguez *et al.* (2022)	2.6%	0.94 [0.63 1.40] (7.57e-01)	0.91 [0.66 1.25] (5.63e-01)	0.87 [0.40 1.92] (7.38e-01)	1.07 [0.78 1.47] (6.92e-01)
**rs56407236**	16:90103687:A:G	*PRDM7*	6.9%	1.11 [1.08 1.14] (6.50e-15)	Bellenguez *et al.* (2022)	6.3%	1.38 [1.06 1.79] (1.67e-02)	1.06 [0.85 1.32] (6.08e-01)	0.79 [0.46 1.35] (3.88e-01)	1.22 [0.99 1.50] (5.80e-02)
**rs35048651**	17:1728046:T:TGAG	*WDR81*	21.4%	1.06 [1.04 1.08] (7.70e-11)	Bellenguez *et al.* (2022)	21.6%	0.95 [0.81 1.10] (4.98e-01)	0.97 [0.86 1.10] (6.29e-01)	0.94 [0.70 1.26] (6.91e-01)	0.97 [0.85 1.09] (5.83e-01)
**rs7225151**	17:5233752:A:G	*SCIMP/ RABEP1*	12.4%	1.08 [1.05 1.10] (4.10e-13)	Bellenguez *et al.* (2022)	12.1%	1.00 [0.82 1.22] (9.97e-01)	1.11 [0.95 1.31] (1.95e-01)	1.19 [0.82 1.72] (3.68e-01)	1.00 [0.86 1.17] (9.88e-01)
**rs12600861**	17:7452302:C:A	*CHRNB1*	35.2%	1.05 [1.04 1.08] (1.01e-08)	Nalls *et al.* (2019)	35.3%	1.00 [0.87 1.14] (9.71e-01)	0.97 [0.87 1.09] (6.32e-01)	1.16 [0.90 1.48] (2.44e-01)	1.01 [0.91 1.13] (8.46e-01)
**rs2242595**	17:18156140:A:G	*MYO15A*	11.2%	0.94 [0.92 0.96] (1.10e-09)	Bellenguez *et al.* (2022)	11.3%	0.87 [0.71 1.06] (1.69e-01)	0.93 [0.80 1.10] (4.01e-01)	0.91 [0.61 1.34] (6.20e-01)	0.91 [0.77 1.07] (2.71e-01)
**rs601999**	17:42546140:T:C	*ATP6V0A1/ PSMC3IP/ TUBG2*	30.1%	1.08 (8.03e-09)	Chang *et al.* (2017)	33.3%	1.07 [0.93 1.23] (3.44e-01)	1.05 [0.94 1.18] (3.76e-01)	1.16 [0.90 1.51] (2.51e-01)	1.01 [0.90 1.13] (9.22e-01)
**rs2269906**	17:44216969:C:A	*UBTF*	34.7%	0.93 [0.92 0.96] (6.24e-10)	Nalls *et al.* (2019)	35.9%	0.97 [0.85 1.11] (6.71e-01)	0.96 [0.87 1.07] (5.17e-01)	0.89 [0.69 1.15] (3.68e-01)	1.00 [0.90 1.11] (9.79e-01)
**rs5848**	17:44352876:T:C	*GRN*	28.9%	1.07 [1.06 1.09] (2.40e-20)	Bellenguez *et al.* (2022)	29.5%	0.98 [0.86 1.13] (8.12e-01)	0.95 [0.85 1.07] (4.00e-01)	1.02 [0.79 1.32] (8.60e-01)	1.01 [0.90 1.13] (8.45e-01)
**rs850738**	17:44357262:G:A	*FAM171A2*	39.4%	1.08 [1.05 1.10] (1.29e-11)	Nalls *et al.* (2019)	40.9%	1.03 [0.91 1.17] (6.49e-01)	0.96 [0.86 1.06] (3.91e-01)	1.02 [0.81 1.30] (8.51e-01)	1.06 [0.96 1.18] (2.57e-01)
**rs17649553**	17:45917282:T:C	*ARHGAP27/ CRHR1/ SPPL2C/ MAPT/ STH/ KANSL1*	23.2%	0.78 [0.76 0.80] (1.26e-68)	Chang *et al.* (2017)	21.2%	0.89 [0.76 1.05] (1.62e-01)	0.91 [0.80 1.03] (1.21e-01)	0.82 [0.60 1.12] (2.06e-01)	0.96 [0.84 1.08] (4.89e-01)

**rs199515**	17:46779275:G:C	*MAPT*	21.9%	0.94 [0.93 0.96] (9.30e-13)	Bellenguez *et al.* (2022)	20.4%	0.93 [0.79 1.09] (3.54e-01)	0.92 [0.81 1.05] (2.00e-01)	0.84 [0.61 1.16] (2.85e-01)	0.96 [0.85 1.10] (5.74e-01)
**rs616338**	17:49219935:T:C	*ABI3*	1.2%	1.32 [1.23 1.42] (2.80e-14)	Bellenguez *et al.* (2022)	1.1%	2.35 [1.02 5.41] (4.47e-02)	1.86 [0.86 4.04] (1.16e-01)	2.64 [0.53 13.00] (2.34e-01)	1.29 [0.80 2.07] (2.89e-01)
**rs2526377**	17:58332680:G:A	*TSPOAP1*	44.5%	0.95 [0.94 0.97] (1.60e-12)	Bellenguez *et al.* (2022)	44.0%	0.91 [0.81 1.04] (1.58e-01)	0.94 [0.85 1.05] (2.62e-01)	1.19 [0.94 1.51] (1.48e-01)	0.95 [0.86 1.06] (3.77e-01)
**rs61169879**	17:61840005:T:C	*BRIP1*	16.4%	1.09 [1.06 1.11] (9.28e-10)	Nalls *et al.* (2019)	15.7%	1.12 [0.94 1.33] (2.14e-01)	1.13 [0.98 1.31] (9.02e-02)	0.92 [0.65 1.31] (6.50e-01)	0.95 [0.83 1.10] (5.02e-01)
**rs4277405**	17:63471557:C:T	*ACE*	38.4%	0.94 [0.93 0.95] (8.80e-20)	Bellenguez *et al.* (2022)	36.7%	1.06 [0.93 1.21] (3.85e-01)	1.06 [0.95 1.19] (2.75e-01)	1.07 [0.84 1.38] (5.70e-01)	0.98 [0.88 1.09] (7.13e-01)
**rs666463**	17:78429399:T:A	*DNAH17*	16.7%	0.93 [0.90 0.95] (3.20e-09)	Nalls *et al.* (2019)	16.6%	1.00 [0.85 1.19] (9.75e-01)	0.97 [0.85 1.12] (7.21e-01)	1.01 [0.73 1.41] (9.46e-01)	1.02 [0.89 1.16] (8.30e-01)
**rs8087969**	18:51157219:G:T	*MEX3C*	45.0%	1.06 [1.04 1.08] (1.41e-08)	Nalls *et al.* (2019)	44.8%	1.03 [0.90 1.17] (6.90e-01)	0.99 [0.89 1.10] (8.90e-01)	0.91 [0.71 1.16] (4.42e-01)	1.03 [0.93 1.14] (5.37e-01)
**rs12151021**	19:1050875:A:G	*ABCA7*	33.6%	1.10 [1.09 1.12] (1.60e-37)	Bellenguez *et al.* (2022)	33.3%	1.23 [1.07 1.42] (3.69e-03)	1.21 [1.08 1.35] (1.18e-03)	1.01 [0.77 1.32] (9.35e-01)	1.02 [0.92 1.14] (6.98e-01)
**rs149080927**	19:1854254:G:GC	*KLF16*	48.0%	1.05 [1.04 1.07] (5.10e-10)	Bellenguez *et al.* (2022)	47.2%	1.03 [0.91 1.17] (5.97e-01)	1.03 [0.93 1.14] (5.94e-01)	1.15 [0.91 1.45] (2.33e-01)	1.04 [0.93 1.15] (5.15e-01)
**rs62120679**	19:2363321:T:C	*LSM7*	32.4%	1.08 [1.05 1.11] (6.64e-07)	Chang *et al.* (2017)	30.2%	0.92 [0.80 1.06] (2.48e-01)	0.98 [0.88 1.10] (7.51e-01)	1.25 [0.96 1.62] (9.53e-02)	1.00 [0.89 1.12] (9.94e-01)
**rs9304690**	19:49950060:T:C	*SIGLEC11*	24.0%	1.05 [1.03 1.07] (4.70e-09)	Bellenguez *et al.* (2022)	25.0%	1.19 [1.03 1.37] (1.87e-02)	1.02 [0.90 1.15] (7.80e-01)	1.05 [0.80 1.40] (7.12e-01)	1.10 [0.98 1.23] (1.13e-01)
**rs587709**	19:54267597:C:T	*LILRB2*	32.5%	1.05 [1.04 1.07] (3.60e-11)	Bellenguez *et al.* (2022)	31.6%	1.10 [0.96 1.26] (1.85e-01)	1.05 [0.93 1.17] (4.43e-01)	0.95 [0.73 1.24] (7.19e-01)	1.05 [0.94 1.17] (4.34e-01)
**rs1358782**	20:413334:A:G	*RBCK1*	24.6%	0.95 [0.94 0.97] (1.60e-08)	Bellenguez *et al.* (2022)	23.7%	1.01 [0.87 1.18] (8.87e-01)	0.94 [0.83 1.07] (3.67e-01)	0.93 [0.70 1.25] (6.47e-01)	1.10 [0.97 1.24] (1.44e-01)
**rs8118008**	20:3187520:G:A	*DDRGK1*	40.4%	1.07 [1.04 1.09] (1.99e-06)	Chang *et al.* (2017)	39.3%	1.05 [0.93 1.20] (4.22e-01)	1.04 [0.93 1.15] (4.97e-01)	0.92 [0.72 1.18] (4.96e-01)	1.03 [0.93 1.14] (5.91e-01)
**rs77351827**	20:6025395:T:C	*CRLS1*	12.8%	1.08 [1.05 1.11] (8.87e-09)	Nalls *et al.* (2019)	11.9%	0.89 [0.73 1.09] (2.51e-01)	0.94 [0.80 1.10] (4.64e-01)	0.94 [0.64 1.38] (7.55e-01)	0.95 [0.81 1.12] (5.49e-01)
**rs6014724**	20:56423488:G:A	*CASS4*	9.0%	0.89 [0.87 0.91] (4.10e-21)	Bellenguez *et al.* (2022)	8.4%	1.00 [0.80 1.24] (9.69e-01)	0.90 [0.75 1.07] (2.30e-01)	0.91 [0.58 1.40] (6.56e-01)	1.05 [0.87 1.26] (6.26e-01)
**rs6742**	20:63743088:T:C	*SLC2A4RG*	22.1%	0.95 [0.93 0.97] (2.60e-09)	Bellenguez *et al.* (2022)	20.6%	0.89 [0.76 1.04] (1.44e-01)	0.93 [0.82 1.06] (2.87e-01)	0.68 [0.48 0.94] (2.05e-02)	0.95 [0.83 1.08] (4.26e-01)
**rs2154481**	21:26101558:C:T	*APP*	47.6%	0.95 [0.94 0.97] (1.00e-12)	Bellenguez *et al.* (2022)	46.9%	1.03 [0.90 1.17] (6.85e-01)	1.05 [0.94 1.17] (3.77e-01)	1.22 [0.96 1.55] (1.11e-01)	0.97 [0.88 1.08] (6.19e-01)
**rs2830489**	21:26775872:T:C	*ADAMTS1*	28.1%	0.95 [0.94 0.97] (1.70e-10)	Bellenguez *et al.* (2022)	28.0%	0.95 [0.82 1.09] (4.30e-01)	0.97 [0.87 1.09] (6.39e-01)	1.08 [0.83 1.40] (5.77e-01)	0.99 [0.89 1.11] (9.19e-01)
**rs2248244**	21:37480059:A:G	*DYRK1A*	28.3%	1.07 [1.05 1.10] (2.74e-11)	Nalls *et al.* (2019)	27.3%	0.98 [0.85 1.14] (8.35e-01)	0.99 [0.88 1.11] (8.33e-01)	0.87 [0.66 1.16] (3.50e-01)	0.98 [0.87 1.10] (7.21e-01)
